# Invasion Pathway of the Ctenophore *Mnemiopsis leidyi* in the Mediterranean Sea

**DOI:** 10.1371/journal.pone.0081067

**Published:** 2013-11-26

**Authors:** Sara Ghabooli, Tamara A. Shiganova, Elizabeta Briski, Stefano Piraino, Veronica Fuentes, Delphine Thibault-Botha, Dror L. Angel, Melania E. Cristescu, Hugh J. MacIsaac

**Affiliations:** 1 Great Lakes Institute for Environmental Research, University of Windsor, Windsor, Ontario, Canada; 2 P.P. Shirshov Institute of Oceanology, Russian Academy of Sciences, Moscow, Russia; 3 Great Lakes Laboratory for Fisheries and Aquatic Sciences, Fisheries and Oceans Canada, Burlington, Ontario, Canada; 4 Dipartimento di Scienze e Tecnologie Biologiche ed Ambientali, Università del Salento, Lecce, Italy; 5 Departament de Biologia Marina i Oceanografia, Institute de Ciencies Del Mar, Barcelona, Catalunya, Spain; 6 Mediterranean Institute of Oceanography, Aix-Marseille Universite, Marseille, France; 7 Recanati Institute for Maritime Studies & Department of Maritime Civilizations, The Charney School of Marine Science, University of Haifa, Haifa, Israel; 8 Biology Department, McGill University, Montreal, Quebec, Canada; The Evergreen State College, United States of America

## Abstract

Gelatinous zooplankton outbreaks have increased globally owing to a number of human-mediated factors, including food web alterations and species introductions. The invasive ctenophore *Mnemiopsis leidyi* entered the Black Sea in the early 1980s. The invasion was followed by the Azov, Caspian, Baltic and North Seas, and, most recently, the Mediterranean Sea. Previous studies identified two distinct invasion pathways of *M. leidyi* from its native range in the western Atlantic Ocean to Eurasia. However, the source of newly established populations in the Mediterranean Sea remains unclear. Here we build upon our previous study and investigate sequence variation in both mitochondrial (Cytochrome *c* Oxidase subunit I) and nuclear (Internal Transcribed Spacer) markers in *M. leidyi*, encompassing five native and 11 introduced populations, including four from the Mediterranean Sea. Extant genetic diversity in Mediterranean populations (*n* = 8, *N*_a_ = 10) preclude the occurrence of a severe genetic bottleneck or founder effects in the initial colonizing population. Our mitochondrial and nuclear marker surveys revealed two possible pathways of introduction into Mediterranean Sea. In total, 17 haplotypes and 18 alleles were recovered from all surveyed populations. Haplotype and allelic diversity of Mediterranean populations were comparable to populations from which they were likely drawn. The distribution of genetic diversity and pattern of genetic differentiation suggest initial colonization of the Mediterranean from the Black-Azov Seas (pairwise *F*_ST_ = 0.001–0.028). However, some haplotypes and alleles from the Mediterranean Sea were not detected from the well-sampled Black Sea, although they were found in Gulf of Mexico populations that were also genetically similar to those in the Mediterranean Sea (pairwise *F*_ST_ = 0.010–0.032), raising the possibility of multiple invasion sources. Multiple introductions from a combination of Black Sea and native region sources could be facilitated by intense local and transcontinental shipping activity, respectively.

## Introduction

Introduction of non-indigenous species (NIS) beyond their native range is considered a principal threat to marine ecosystems worldwide [1]. The rate of such introductions accelerated in the past few decades in conjunction with increased maritime shipping and global trade [2]–[3]. Maritime traffic often involves use of ballast water loaded in source ports and later discharged in destination ports, resulting in mass transfer of organisms between distant regions [4]–[6]. Species with planktonic life stages have a high chance of interfacing with a shipping vector when ballast water is loaded, and thus of being moved around the world to new locations [7].

In recent years, gelatinous zooplankton outbreaks have raised concerns regarding the health of aquatic ecosystems [8]. A number of biological traits of gelatinous zooplankton may contribute to global outbreaks by this group. For example, many gelatinous zooplankton have a broad diet, high growth rate, high fecundity, high regeneration, encystment, and even reverse development potential [9]–[11], which enable them to overcome harsh conditions associated with the transport vector (i.e. ballast tanks) and successfully reach and establish in new environments [12]–[14].

The Mediterranean Sea has an enormously rich native biodiversity, though it is also the world's most invaded marine ecosystem [15]–[16] and is considered at very high risk of future invasions from ballast water discharges [17] and, especially, canal connections [18]–[19]. A total of 986 NIS have been recorded in the Mediterranean Sea, including 48 new species since 2011 [19]. The eastern section of the Sea has accumulated a disproportionate number of these NIS, principally due to Lessepsian invaders [18]–[20] that colonized following opening of the Suez Canal with its link to the Indian Ocean.

Knowledge of the source and pathways of NIS introductions is essential for developing management strategies to prevent invasions. A focus on areas at high risk of biological invasions is crucial and should be considered a management priority [17], [21]–[22]. In this paper, we explore the spread of the ctenophore *Mnemiopsis leidyi* A. Agassiz 1865 to the Mediterranean Sea. *Mnemiopsis leidyi* is native to the western Atlantic Ocean from Massachusetts, USA to Argentina. The species is a simultaneous hermaphrodite capable of self-fertilization, may reach maturity at two weeks of age, and can release up to 10,000 eggs per day [23]. Over the past 30 years, the species spread across Europe in a remarkable series of invasions, first entering the Black Sea (and Azov Sea) in early 1980s [24], the eastern Mediterranean in early 1990s (mainly Aegean Sea where an established population was not reported, [25]–[26]), followed by the Caspian Sea in 1999 [27].

Blooms of *M. leidyi* were reported throughout the Mediterranean Sea in 2009, from eastern to western coastal areas [28]–[31]. Previous studies have addressed invasion pathways of *M. leidyi* from its native region to Eurasia excepting the Mediterranean Sea [32]–[33]. These studies suggested that *M. leidyi* was introduced to Eurasia via at least two pathways. The first invasion occurred from the Gulf of Mexico to the Black Sea, followed by secondary spread to the Caspian Sea [32]–[33]. The second invasion was from the northern distribution of this species in the western Atlantic (possibly Narragansett Bay) to the Baltic and North Seas in northern Europe [32]–[33]. However, the source of the *M. leidyi* population in the Mediterranean Sea remains unclear. Several possibilities can be envisaged. It is possible the species has spread exclusively from the Black Sea [34] or other south Eurasian Seas in currents or in discharged ballast water. Alternatively, the species may have spread in discharged ballast water that originated in the North or Baltic seas, from the western Atlantic Ocean, or via a combination of the above pathways. To clarify the invasion pathway(s) of this species into the Mediterranean Sea, here we explore the population genetic structure of native and introduced populations using both mitochondrial (Cytochrome *c* Oxidase subunit I; COI) and nuclear ribosomal (Internal Transcribed Spacer; ITS) genes.

## Materials and Methods

### Ethics Statement

No specific permits were required for the described field studies in Eurasia, North America or South America. The species collected is an invasive pest in Eurasia and is not protected throughout its range. Sampling points did not include any protected or private lands.

### Sample Collection and DNA Extraction

A total of 286 *M. leidyi* individuals were sampled from five native (Narragansett Bay, Massachusetts; York River, Virginia; Morehead, North Carolina; Tampa Bay, Florida; Peninsula Valdes coast, Argentina) and 11 introduced populations (two from the eastern Black Sea; Sea of Azov; north and south Caspian Sea; Baltic Sea; Limfjorden Fjord, Denmark; and Spain, France, Italy and Israel in the Mediterranean Sea). Individuals were preserved separately in 95% ethanol prior to genetic analysis.

Genomic DNA was isolated from gelatinous lobe tissue of the ctenophores using the automatic extraction protocol described by Elphinstone et al. [35], and DNeasy Blood and Tissue Kit (Qiagen Inc., ON, Canada). A fragment of the COI gene was amplified using the species-specific primers (Ml-COIF: 5′- TGTCGCCCAAATTACTGTTTC-3′ and Ml-COIR: 5′- TGACGGGGTAAACCTCATAAA-3′). Primers were designed in this study according to the available sequenced *M. leidyi* mitochondrial genome (GenBank accession no: NC016117). The universal primer pair, (ITS5F and ITS4R) [36] was used to amplify the ITS-1, 5.8 S gene, and ITS-2. We conducted PCR amplifications in a 40-µL reaction volume, with about 50 ng of genomic DNA, 1 unit of *Taq* DNA Polymerase (QIAGEN), 1 x PCR buffer, 2.5 mM of MgCl_2_, 0.2 mM of dNTPs, and 0.4 µM of each primer. PCR was performed with an initial denaturing step at 95°C for 1 min, followed by 35 amplification cycles (95°C for 30 s, 50°C for 30 s, 72°C for 50 s), and a final elongation step at 72°C for 7 min.

### Sequencing and Cloning Protocol

We purified PCR products, which were then sequenced for both COI and ITS markers with forward (Ml-COIF) and reverse primers (ITS4R), respectively, using Big Dye terminator sequencing chemistry with an ABI 3130XL genetic analyzer (Applied Biosystems). Sequences were inspected, manually edited, and aligned using Codon Code Aligner 2.0 (Codon Code Corporation, Dedham, MA). Sequence of alleles containing double nucleotide calls (overlapping peaks) were cloned using Cloning and Amplification Kit (pSMART GC HK, Lucigen) according to Ghabooli et al. [33].

### MtDNA Analysis

We assessed diversity indices within populations, such as the number of haplotypes (*n*), haplotype diversity (*h*) and nucleotide diversity (*π*) [37] using DnaSP v5 [38]. We constructed phylogenetic relationships among haplotypes using the neighbor-joining algorithm in MEGA version 4 [39]. We used a fragment of COI from a cydippid ctenophore, *Pleurobrachia pileus* (GenBank accession no JF760211) as an outgroup. We generated a parsimony network of haplotypes using TCS 1.0 [40].

### Nuclear marker (ITS) Analysis

Using the protocol described above, we processed four new populations from Mediterranean Sea (Spain, France, Italy and Israel) as well as one more from the native range (MH from North Carolina) in addition to our previously published dataset which consisted of 190 individuals analyzed for ITS marker [33]. We measured genetic diversity within populations with number of alleles (*N_a_*), observed (*H_o_*), and calculated expected heterozygosity (*H_e_*) using GENEPOP (online version http://genepop.curtin.edu.au) and Arlequin version 3.1 [41]. We used the Markov chain method to estimate the probability of significant deviation from Hardy-Weinberg equilibrium using GENEPOP. We determined genetic differentiation among populations from pairwise *F*_ST_ using Arlequin.

To estimate the sufficiency of our sampling, we generated rarefaction curves using ECOSIM and 5000 random iterations [42] for both haplotypes and alleles found in native region, and the Black-Azov and Mediterranean Seas. We estimated Chao-1 diversity [43] using SPADE software version 3.1 [44], based on the number of rare haplotype/allele present in sampled populations.

## Results

Analysis of a 656-bp fragment of COI obtained from 241 individuals resulted in 17 different haplotypes in surveyed populations (GenBank accession nos KF435105–KF435121). In total, we detected 29 variable sites (4.42%), 16 of which were specific to the divergent haplotype Ml01 from Peninsula Valdes, Argentina (2.43%). Ml03 and Ml09 were the most common haplotypes. We found haplotype Ml03 in all populations except in Peninsula Valdes, while Ml09 was not recovered from Peninsula Valdes, Limfjorden, or the Baltic Sea.

**Figure 1:**
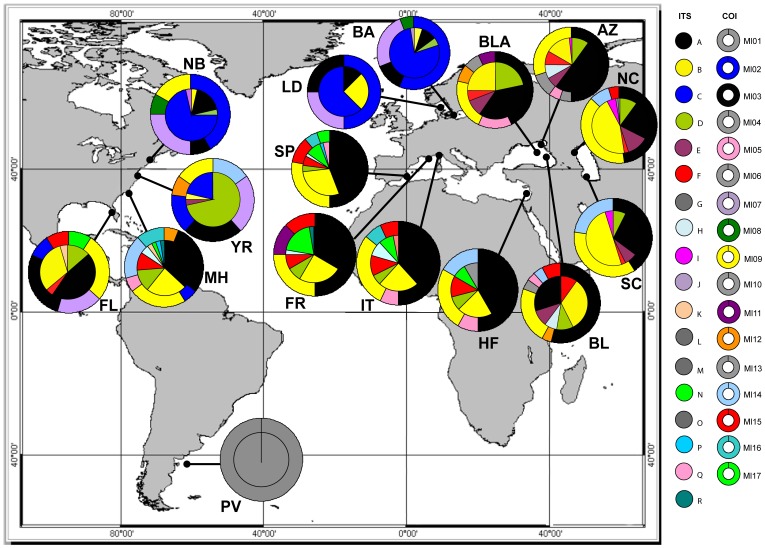
Haplotype distribution and frequency map for *Mnemiopsis leidyi*. Allele (inner circle for ITS) and haplotype (outer donut for COI) distribution map of *Mnemiopsis leidyi*. Each color indicates a different allele/haplotype. Private alleles/haplotypes are highlighted in grey. Population codes are described in Table 1.

We found twelve different haplotypes in native populations, all of which were present in introduced populations except for Ml01 from Peninsula Valdes, the single private haplotype at this site. We detected a total of 16 haplotypes among the introduced populations. Black-Azov Sea populations contained 11 haplotypes, which was higher than in all other introduced regions. Mediterranean Sea populations contained eight haplotypes, while those from the Caspian and Baltic seas had four haplotypes each. Out of eight haplotypes observed in the Mediterranean Sea, only Ml11 was not recovered from native populations in North America. Six haplotypes including Ml11 were detected in Black-Azov Seas. Two haplotypes from Mediterranean Sea populations were not found in either the Black or Azov Sea, though they were present in the native region, mainly in Florida and Morehead (Figure 1, Table 1).

**Table 1:** Population code, sample size (*N*), number of haplotypes (*n*), haplotype diversity (h), nucleotide diversity (*π*), number of alleles (*N_a_*), observed (*H_o_*) and expected (*H_e_*) heterozygosity, and P-value for Hardy-Weinberg equilibrium (HWE) analysis; bold numbers correspond to populations deviating significantly (P<0.05) from HWE.

ID	Collection site	Collection date	mtDNA					Internal Transcribed Spacer (ITS)				
			*N*	*n*	Haplotype Code	*h*	*π*	*N*	*N_a_*	*H*_O_	*H*_E_	HWE *P*-<
Introduced												
AZ	Seaof Azov, Yasenskaya Bay	2006	20	5	Ml03–06, Ml09	0.679	0.0020	30	7	0.70	0.70	0.892
BL	Black Sea, transect from Blue Bay	2007	26	7	Ml03, Ml05, Ml09–10, Ml12, Ml14–15	0.671	0.0019	20	6	0.55	0.76	**0.002**
BLA	Black Sea, near Gelendzhik	2007	14	6	Ml03, Ml05, Ml09, Ml11–13	0.791	0.0021	16	5	0.62	0.76	**0.003**
NC	North Caspian Sea, Makhachkala coast	2007	23	4	Ml03, Ml09, Ml14–15	0.636	0.0019	40	8	0.65	0.73	**0.007**
SC	South Caspian Sea, Sari and Noor coasts	2007	22	3	Ml03, Ml09, Ml14	0.680	0.0020	20	6	0.50	0.70	**0.009**
BA	Baltic Sea, Kiel,Germany	2007	16	4	Ml02–03, Ml07–08	0.642	0.0018	20	5	0.30	0.39	0.086
MD	Limfjorden Fjord, Denmark	2011	4	3	Ml02–03, Ml07	0.833	0.0030	4	3	0.50	0.60	0.431
SP	Dénia, Spain	2010	18	5	Ml03, Ml09, Ml15, Ml16, Ml17	0.693	0.0019	26	8	0.54	0.72	**0.000**
FR	Berre Lagoon, Marseille, France	2010	16	4	Ml03, Ml09, Ml11, Ml15	0.700	0.0019	18	7	0.55	0.80	**0.000**
IT	Ligurian Sea, Italy	2010	14	5	Ml03, Ml05, Ml09, Ml15–16	0.703	0.0020	17	7	0.65	0.79	0.061
HF	Haifa, Israel	2010	12	4	Ml03, Ml05, Ml09, Ml14	0.712	0.0019	12	6	0.58	0.78	0.080
Native												
NB	Narragansett Bay, RI	2008	12	5	Ml02–03, Ml07–09	0.788	0.0037	14	5	0.50	0.47	0.568
YR	York River, VI	2008	13	6	Ml02–03, Ml07, Ml09, Ml12, Ml14	0.885	0.0043	14	4	0.57	0.45	0.733
MH	Morehead, NC	2010	17	7	Ml02–03, Ml05, Ml09, Ml12, Ml14, Ml16	0.853	0.0028	19	9	0.76	0.82	0.079
FL	Tampa Bay, FL	2006	11	6	Ml02–03, Ml07, Ml09, Ml15, Ml17	0.873	0.0042	11	5	0.63	0.76	0.554
PV	Peninsula Valdes, Argentina	2009	3	1	Ml01	0.000	0.0000	5	1	0.00	0.00	0.000
Total			241					286				

The Black-Azov Seas shared six haplotypes with native populations, while the other five haplotypes from this region were either private for one population (Ml04, Ml05, Ml10, and Ml13) or shared with France in Mediterranean Sea (Ml11). All four haplotypes found in Caspian Sea populations were present in both the Black Sea and North America. The Baltic Sea and Limfjorden (Denmark) shared all of their haplotypes with the native region, mainly Narragansett Bay, and only one haplotype with other introduced populations (Figure 1, Table 1).

The introduced population (BL) from Black Sea contained the highest number of haplotypes (*n* = 7) (Table 1). Among introduced populations, those from Limfjorden and the south Caspian Sea had the lowest number of haplotypes (*n* = 2 and 3, respectively). Native populations from Morehead and Peninsula Valdes exhibited the highest (*n* = 7) and lowest (*n* = 1) number of haplotypes, respectively (Table 1).

Mean COI haplotype diversity (*h*) and nucleotide diversity (*π*) in all introduced populations were 0.704±0.059 and 0.0020±0.0003, respectively. Comparable values in Mediterranean Sea populations were nearly identical, 0.702±0.008 and 0.0019±0.0001, respectively. Native populations exhibited higher values for each of these indices (*h* = 0.850±0.043 and *π* = 0.0038±0.0007, respectively). We excluded the non-diverse individuals of Peninsula Valdes of South America from this calculation.

**Figure 2:**
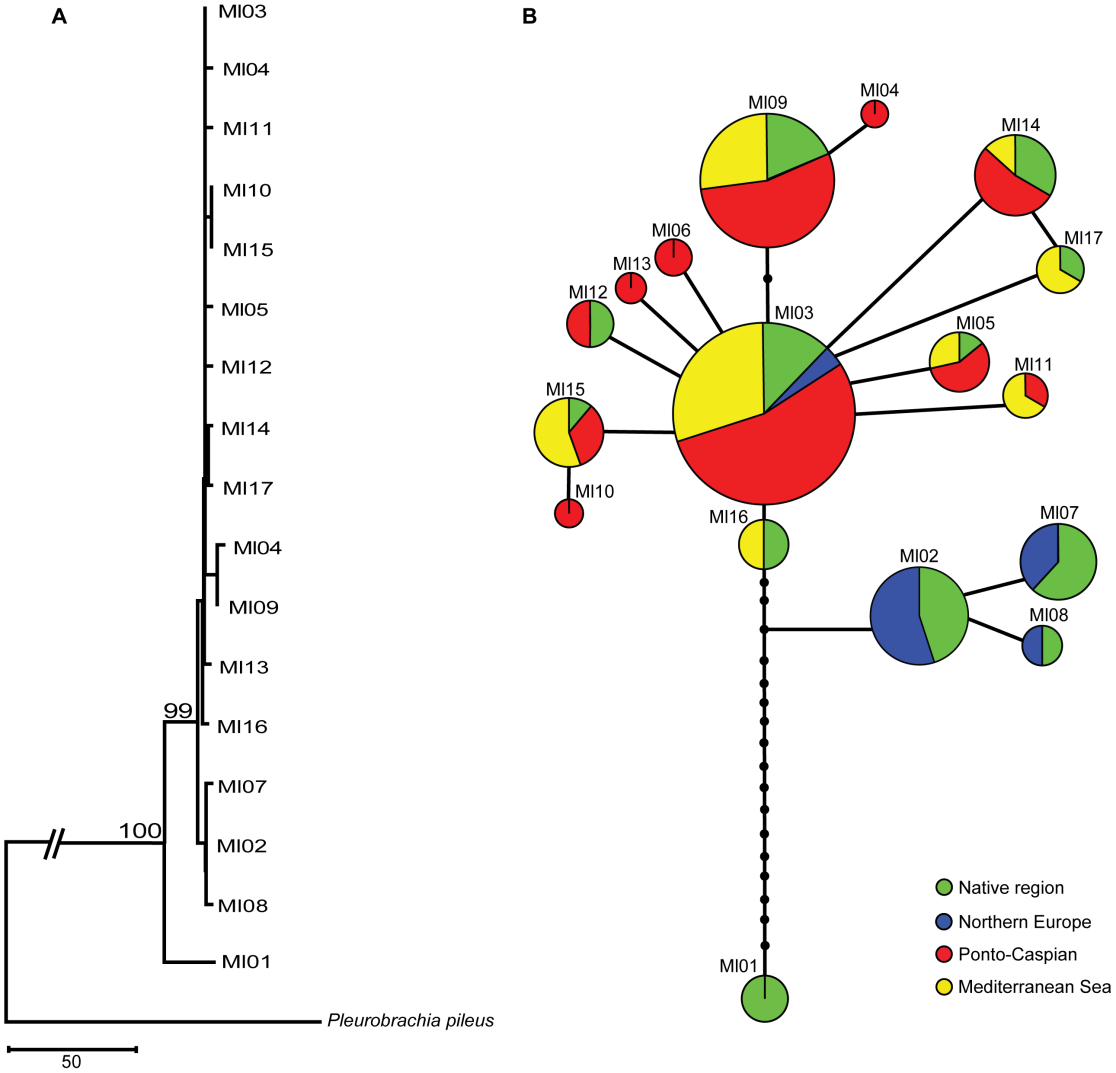
Phylogenetic analyses of *Mnemiopsis leidyi*. Phylogenetic and network relationship between the 17 haplotypes identified in the alignment of COI (A) Neighbor joining phylogenetic tree based on nucleotide divergence calculated using Tamura-Nei model. (B) Network relationships among haplotypes for native and introduced populations, inferred by statistical parsimony. Pie charts correspond to sampled haplotypes described in Figure 1. The size of the charts corresponds to the frequency of the haplotype among all samples. Black circles indicate missing haplotypes and each line represents a single mutation step. Colours show different locations for recovered haplotypes: green: native region, blue: Northern Europe, red: Ponto-Caspian region, and yellow: Mediterranean Sea.

The reconstructed phylogenetic relationship for the mtDNA haplotypes supported three main groups. The first group consists of the unique and highly divergent Ml01 haplotype restricted to South America, whereas the second one includes haplotypes Ml02, Ml07 and Ml08, which were common in northern areas of the distribution range in North America and Europe (Narragansett Bay, Baltic Sea and Limfjorden). The rest of the haplotypes formed the third group (Figure 2A). The complex parsimony haplotype network was star-shaped for the third group, with Ml03 in the middle. There were one or a few mutation steps between haplotypes, except for Ml01, which was separated from Ml03 by 19 mutation steps (Figure 2B).

**Figure 3:**
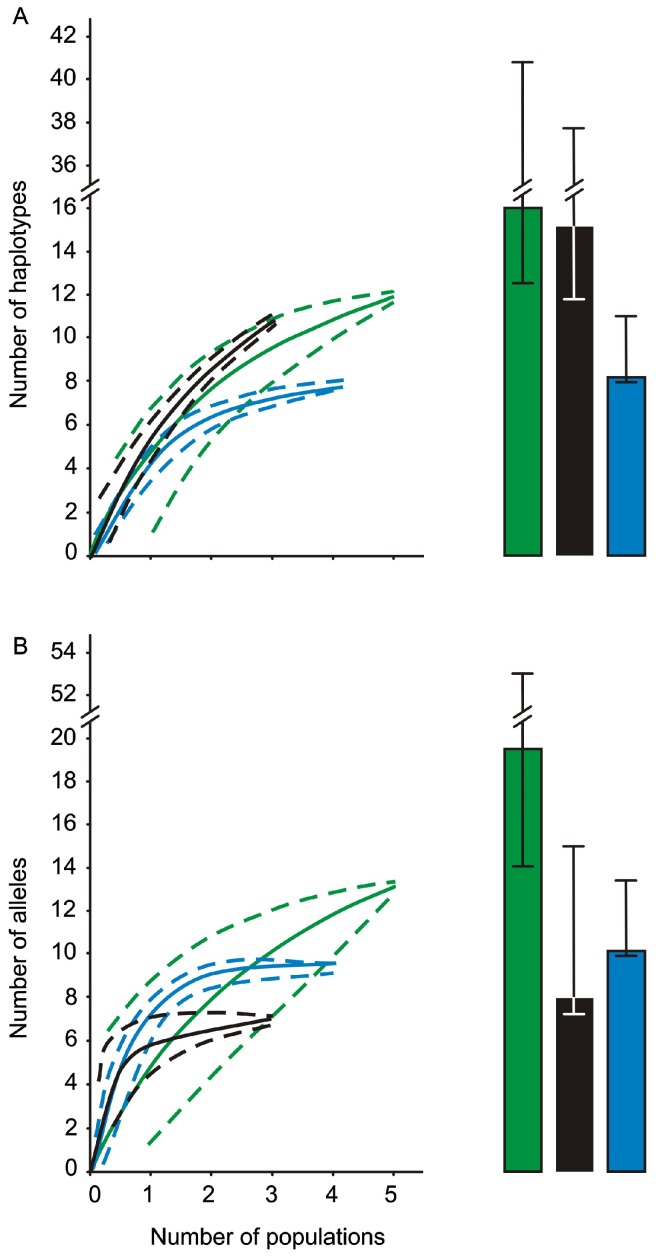
Rarefaction curves and Chao-1 estimates for Mediterranean Sea and putative source populations. Sample-based rarefaction curves of native populations (green line, ±95% C.I.), Black-Azov Seas (black line, ±95% C.I.), and Mediterranean Sea (blue line, ±95% C.I.) for (A) COI haplotypes and (B) ITS alleles found in surveyed *Mnemiopsis leidyi* populations. Estimates of haplotype and allele richness (Chao-1, ±95% C.I.) are shown in each panel for native populations (green bar), Black-Azov Seas (black bar), and Mediterranean Sea (blue bar). Note the break in the y-axis scale.

Chao-1 COI haplotype richness estimates were moderately higher than obtained values in Black-Azov Sea populations (15.2 vs. 11, respectively), indicating undersampling of these regions, although the lower 95% confidence interval limit (11.7) was marginally higher than observed diversity in the Black-Azov Seas (Figure 3A). Chao-1 estimates for native region were also higher than the observed diversity (16 vs. 12), with the lower 95% confidence interval limit of 12.6 suggesting moderate undersampling of native region (Figure 3A). However, Chao-1 estimates for the Mediterranean Sea were similar to the observed diversity (8.1 vs. 8), with the lower 95% confidence interval limit of 8 suggesting sampling was sufficient (Figure 3A). The percentage of singletons for the Chao analyses of the native region, Black-Azov and Mediterranean Seas was 33, 45, and 13, respectively.

Analysis of the 619 bp DNA fragment comprising the complete ITS1, 5.8 S rRNA and ITS2 regions obtained from 286 individuals of *M. leidyi* - including the 190 individuals analyzed in our previous study [33] - resulted in 18 different alleles. We found five new alleles (GenBank accession nos KF435100–KF435104) in the Mediterranean Sea and Morehead (Figure 1) which were not previously identified. Alleles N and O were the most and least common, respectively. Alleles A and B were the most common in all populations (Figure 1), consistent with the previous survey of Ghabooli et al. [33].

We detected thirteen different alleles in native populations, all of which were recovered from introduced populations, except for the private allele G from Peninsula Valdes (Figure 1). Mediterranean Sea populations had 10 alleles, eight of which were present in native region. There was one private allele (O) in Haifa, Israel (Figure 1). Only five of 10 alleles found in Mediterranean populations were shared with Black-Azov Sea populations. In total, we recovered seven alleles in the Black-Azov Seas, six of which were also obtained from North America. Alleles C and J in Baltic Sea, Limfjorden, and Narragansett Bay were not present in Mediterranean populations, consistent with Black, Azov and Caspian Seas (Figure 1, Table 1, see Ghabooli et al. [33]).

The Chao-1 allele richness estimate for the Black-Azov Seas (Chao-1 estimator  = 8; lower 95% confidence interval  = 7.1; allele richness  = 7) indicates reasonably comprehensive sampling of this region (Figure 3B). For the native region, the estimated Chao-1 allele richness was 19.3, while the observed richness was 13, indicating undersampling of this area (Figure 3B). For Mediterranean Sea populations, the Chao-1 estimates were similar to the observed diversity (10.2 vs. 10) with the lower 95% confidence interval of 10 indicating sufficient sampling in this region (Figure 3B). The percentage of singletons for the native region, and Black-Azov and Mediterranean Seas was 38, 14, and 10, respectively.

Mean observed heterozygosity (*H_o_*) was lower in introduced populations (0.56±0.011) than in native ones (0.62±0.12) (Table 1). Pairwise *F*_ST_ values in Mediterranean populations ranged from 0.011 to 0.033. All populations had highest *F*_ST_ values with Peninsula Valdes, Argentina due to fixation of a private and divergent allele in the South American population (Table 2).

**Table 2:** Population subdivision according to pairwise *F*_ST_ values.

	AZ	BL	BLA	NC	SC	BA	LD	SP	FR	IT	HF	NB	YR	MH	FL
**BL**	0.008														
**BLA**	0.004	0.019													
**NC**	**0.087**	0.008	0.030												
**SC**	**0.074**	0.005	0.023	0.017											
**BA**	**0.398**	**0.383**	**0.392**	**0.384**	**0.417**										
**LD**	**0.248**	**0.182**	**0.203**	**0.200**	**0.199**	0.015									
**SP**	0.007	0.006	0.009	**0.053**	0.031	**0.395**	**0.211**								
**FR**	0.028	0.002	0.007	**0.049**	0.035	**0.374**	**0.177**	0.011							
**IT**	0.001	0.017	0.001	**0.051**	0.034	**0.380**	**0.187**	0.020	0.023						
**HF**	0.005	0.016	0.013	**0.058**	0.040	**0.397**	**0.192**	0.023	0.026	0.033					
**NB**	**0.330**	**0.317**	**0.322**	**0.337**	**0.358**	0.022	0.021	**0.327**	**0.306**	**0.308**	**0.319**				
**YR**	**0.350**	**0.319**	**0.263**	**0.321**	**0.363**	**0.469**	**0.411**	**0.370**	**0.316**	**0.337**	**0.341**	**0.437**			
**MH**	0.001	0.021	0.020	**0.041**	0.026	**0.369**	**0.181**	0.010	0.013	0.025	0.029	**0.300**	**0.291**		
**FL**	0.008	0.037	0.028	0.015	0.005	**0.408**	**0.190**	0.015	0.013	0.024	0.032	**0.335**	**0.324**	0.036	
**PV**	**0.503**	**0.479**	**0.496**	**0.478**	**0.514**	**0.708**	**0.720**	**0.494**	**0.466**	**0.477**	**0.502**	**0.681**	**0.692**	**0.466**	**0.522**

Significant differences are bolded. Population codes correspond to Table 1.

Introduced populations from the Mediterranean Sea had the lowest *F*_ST_ values with those from the Black and Azov Seas (*F*_ST_ = 0.001–0.028). However, Mediterranean Sea populations were also very similar to those from the Gulf of Mexico (FL) and North Carolina (MH) in the native region (*F*_ST_ = 0.010–0.032; Table 2). Within the Mediterranean Sea, populations from Spain and France had the lowest *F*_ST_ value (0.011), while those in Italy and Israel were most divergent (*F*_ST_ = 0.033). Individuals from Limfjorden, Denmark had the lowest *F*_ST_ with Baltic Sea (*F*_ST_ = 0.015), and with Narragansett Bay (*F*_ST_ = 0.021) in the native region (Table 2).

## Discussion

In this study, we build upon our previous study to explore genetic diversity, and determine the source(s) of, *Mnemiopsis leidyi* populations in the Mediterranean Sea using both mitochondrial (COI) and nuclear (ITS) markers. Our results support a multiple source model, composed by at least two different introduction pathways. One source of *M. leidyi* in the Mediterranean appears to have originated from Black Sea, consistent with the view of Bolte et al. [34] and with natural flows between the basins. However, we propose a second possible invasion pathway, originating from North America (Gulf of Mexico).

### Genetic diversity and population differentiation

Introduced populations in the Mediterranean Sea exhibited lower values of haplotype diversity (0.702±0.008) and observed heterozygosity (0.58±0.05) relative to native ones (0.850±0.043 and 0.62±0.19, respectively). However, none of the Mediterranean populations exhibited erosion of genetic diversity for either of the analyzed markers relative to their putative source populations. This pattern could be driven by repeated introductions from the native range as well as from the adjacent Black Sea area, given intense vector activity between these regions and the high diversity of source populations [17], [45].

Two Mediterranean Sea populations (Spain and France) exhibited deviation from Hardy-Weinberg equilibrium (Table 1). Both populations exhibited lower than expected heterozygosity, which can be explained by possible inbreeding and/or population admixture (i.e. Wahlund effect) [33], [46]. We did not detect a heterozygosity deficit in other newly analyzed populations in Morehead and Limfjorden (Table 1).

Mediterranean populations had the lowest *F*_ST_ with populations from the Black-Azov Seas (*F*_ST_ = 0.001–0.028). However, Mediterranean populations also exhibited low genetic differentiation with those from Florida and Morehead (*F*_ST_ = 0.010–0.032) in the native range. The highest genetic differentiation occurred among introduced populations in Mediterranean or Black-Azov-Caspian seas and those in the Baltic Sea and Limfjorden (Denmark), ranging from 0.177 to 0.417 (Table 2). High genetic divergence between introduced populations implies very low or lack of genetic connectivity and gene flow among these locations, implying that northern populations were not responsible for invasion of the Mediterranean Sea. As well, initial reports of invasion of the Mediterranean Sea occurred prior to those from the Baltic or North Seas [25]–[26].

**Figure 4:**
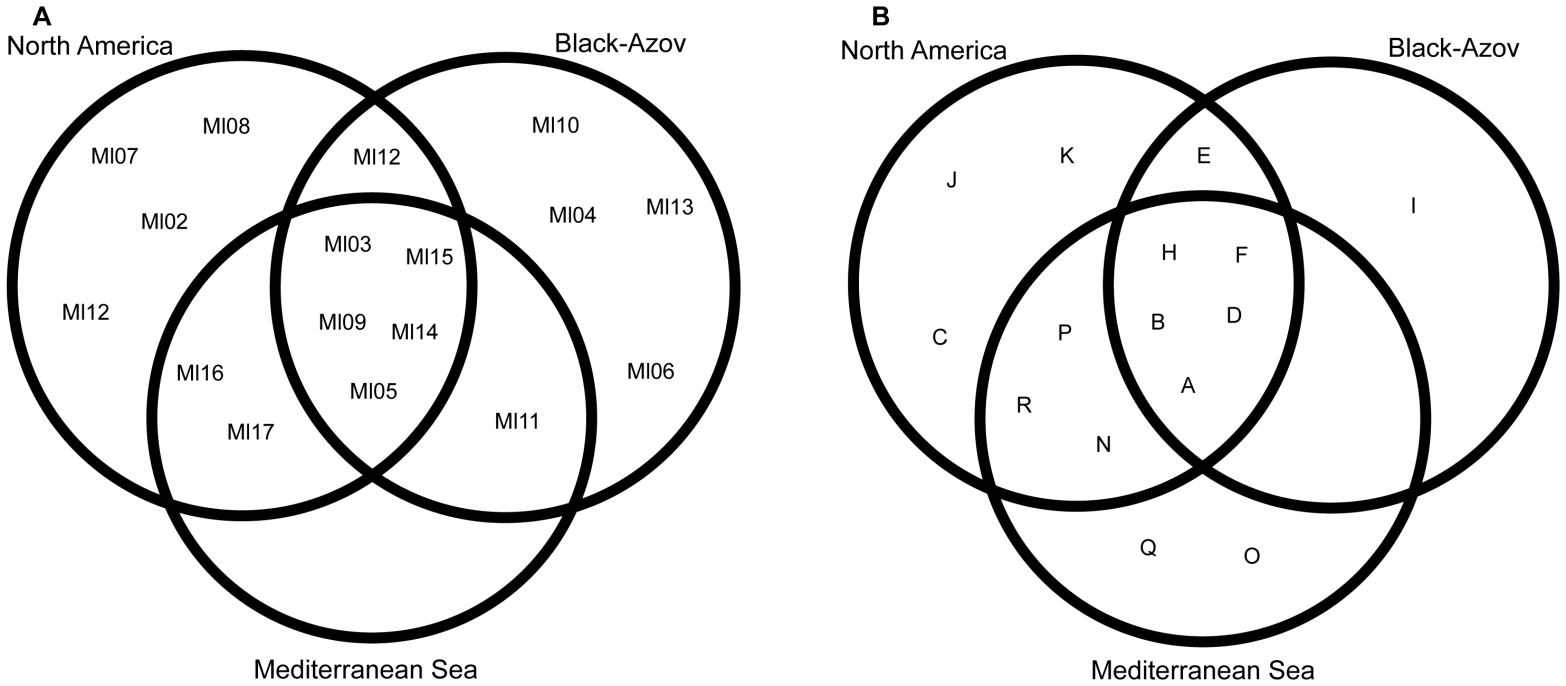
Venn diagram illustrating shared haplotypes/alleles between regions. Venn diagram showing COI haplotypes (A) and ITS alleles (B) sharing between Mediterranean and possible source populations from North America and Black-Azov Seas. Note that haplotype Ml01 and allele G from South America are excluded.

Our results suggest that the Black-Azov Seas are a likely source of *M. leidyi* in the Mediterranean Sea, in accordance with Bolte et al. [34]. It is important to note, however, that the presence of similar alleles and haplotypes in the Mediterranean Sea and native populations - specifically those in the Gulf of Mexico and North Carolina - suggest a possible invasion pathway from North America. Namely, two COI haplotypes (Ml16 and Ml17) found in Mediterranean populations were not recovered from Black or Caspian Seas, but were present in native populations (Figure 4A) in North America (Florida and Morehead). Similarly, our ITS survey revealed five new alleles for this species which were not recovered from populations in Sea of Azov, Black or Caspian Seas (Figure 4B). Although the absence of the above alleles/haplotypes in Black and Caspian Seas populations may be explained by insufficient sampling from these regions or by seasonal, variation in frequency of genotypes/haplotypes, or other ecological and evolutionary processes, the possibility of introduction of *M. leidyi* from the native source region cannot be excluded. This conclusion is supported by our Chao-1 diversity estimates and rarefaction curves for native and Black-Azov Seas populations. These analyses indicate that our sampling recovered most of the diversity present in native and especially in the Black-Azov Seas and, therefore, the Black Sea as a sole source seems less likely.

The Mediterranean Sea receives an enormous flow of global shipping [16]–[17]. The tropical Western Atlantic Ocean is a source of trade to the Mediterranean Sea, and places it at risk of future invasions from discharged ballast water [17]. Moreover, high shipping activity within the Mediterranean Sea itself poses additional risk of translocation of *M. leidyi* and other NIS throughout the basin [17].

Despite of *M. leidyi*'s dynamic invasion history, we observed geographic structure with some haplotypes/alleles being restricted to particular latitudes. The geographic distribution of genetic diversity is clearly not random and appears to reflect adaptation to specific biogeographic conditions. It is likely that this association is not only due to vector directionality but also to ecological and evolutionary processes [47]. The three haplotypes forming the second group in the NJ tree are very common in the northern region and less prevalent elsewhere (Figure 2A). The rest of the haplotypes that form the star in the parsimony network are distributed mainly in warmer waters and some were not found at all in northern regions (Figure 2B). Shifts in haplotype/allele frequencies are expected due to selection to local conditions. Some haplotypes/alleles could become dominant in several generations if they are strongly favored by selection or linked to regions favored by selection [48]–[49].

Genetic differentiation among native populations was relatively high (*F*_ST_ = 0.036–0.437), suggesting some structuring and limited gene flow in the native region. The private haplotype Ml01 was separated from other haplotypes by at least 15 mutation steps. All other haplotypes had only one or a few mutation steps between them. Pairwise genetic differentiation, parsimony network analysis, and phylogenetic reconstruction of haplotypes demonstrate high genetic divergence between South America and all other locations, notwithstanding the paucity of samples available from the former region. Long-term isolation of populations could explain the observed divergence. Pleistocene glacial periods in the northern hemisphere could drive high genetic divergence between South America and North America, resulting in population fragmentation [50]. However, further studies and more comprehensive sampling of the region, especially South and Central America, could shed light on the degree of isolation between populations along the western Atlantic coast. Our present, albeit very limited analysis does not support an introduction pathway for *M. leidyi* between South America and Eurasia.

### Introduction pathways

Genetic analyses have revealed pathways of *M. leidyi* introduction into major Eurasia Seas [32]–[34]. *M. leidyi* entered the Black Sea via ships' ballast water from the Gulf of Mexico region. Spread of *M. leidyi* into the Sea of Azov occurred via the natural connection between these basins [26]. Secondary introduction into Caspian Sea likely occurred through ballast water discharged by a vessel after transiting the Volga-Don canal [51]. A second pathway from a port in New England, possibly Narragansett Bay, was likely responsible for the translocation of *M. leidyi* into the Baltic Sea, with subsequent spread into the North Sea [32]. The Mediterranean Sea was the most recent European basin invaded, with the eastern portion of the basin colonized first. Water flow between the Black and Mediterranean seas could account for this invasion, with subsequent transfer within the latter accommodated by a combination of ballast transfer and natural spread. Bolte et al. [34] used six microsatellite data to suggest a Black Sea source of *M. leidyi* in the Mediterranean Sea. However, in this study, genetic differentiation of North American and Mediterranean Sea populations was only slightly greater than that with populations from the Black Sea (Table 2). In addition, there were more haplotypes/alleles present in the Mediterranean Sea that were not shared by Black-Azov populations than with those from the Gulf of Mexico region (Table 1). Finally, there exists substantial ballast water movement from the Gulf region to the Mediterranean Sea [17]. Each of these lines of evidence supports the view that North America could have been an additional source of the introduced population in the Mediterranean Sea. The analysis of ITS and COI data in this study are consistent with the hypothesis of multiple introductions, with both native and Black Sea populations serving as sources of *M. leidyi* in the Mediterranean Sea.

## References

[1] Molnar JL, Gamboa RL, Revenga C, Spalding MD (2008) Assessing the global threat of invasive species to marine biodiversity. Frontiers Ecol Environ 6: 485–492.

[2] Hulme PE (2009) Trade, transport and trouble: managing invasive species pathways in an era of globalization. J Appl Ecol 46: 10–18.

[3] Ruiz GM, Fofonoff PW, Carlton JT, Wonham MJ, Hines AH (2000) Invasion of coastal marine communities in North America: apparent patterns, processes, and biases. Ann Rev Ecol System 31: 481–531.

[4] Ruiz GM, Carlton JT, Grosholz ED, Hines AH (1997) Global invasions of marine and estuarine habitats by non-indigenous species: mechanisms, extent, and consequences. Integr Comp Biol 37: 621–632.

[5] Ruiz GM, Carlton JT (2003) Invasion vectors: a conceptual framework for management. In: Ruiz GM, Carlton JT, editors. Invasive species, vectors and management strategies. Island Press, Washington DC. pp. 459–498.

[6] Briski E, Bailey SA, Casas-Monroy O, DiBacco C, Kaczmarska I, et al (2013) Taxon- and vector-specific variation in species richness and abundance during the transport stage of biological invasions. Limnol Oceanogr 58: 1361–1372.

[7] Rejmanek M, Richardson DM (1996) What attributes make some plants more invasive? Ecology 77: 1655–1661.

[8] Link JS, Ford MD (2006) Widespread and persistent increase of Ctenophora in the continental shelf ecosystem off NE USA. Mar Ecol Prog Ser 320: 153–159.

[9] Boero F, Belmonte G, Fanelli G, Piraino S, Rubino F (1996) The continuity of living matter and the discontinuities of its constituents: do plankton and benthos really exist? Trends Ecol Evol 11: 177–180. 21237800 10.1016/0169-5347(96)20007-2

[10] Boero F, Bouillon J, Piraino S, Schmid V (2002) Asexual reproduction in Hydrozoa (Cnidaria). In: R.N Hughes, editor. Reproductive Biology of Invertebrates - Progress in Asexual Reproduction New Delhi, Oxford & IBH Publishing. pp.141–158.

[11] Piraino S, De Vito D, Schmich J, Bouillon J, Boero F (2004) Reverse development in Cnidaria. Can J Zool 82: 1748–1754.

[12] Boero F, Bouillon J, Gravili C, Miglietta MP, Parsons T, et al (2008) Gelatinous plankton: irregularities rule the world (sometimes). Mar Ecol Progr Ser 356: 299–310.

[13] Richardson AJ, Bakun A, Hays GC, Gibbons MJ (2009) The jellyfish joyride: causes, consequences and management responses to a more gelatinous future. Trends Ecol Evol 24: 312–322. 19324452 10.1016/j.tree.2009.01.010

[14] Piraino S, Fanelli G, Boero F (2002) Variability of species' roles in marine communities: change of paradigms for conservation priorities. Mar Biol 140: 1067–1074.

[15] Galil BS (2007) Loss or gain? Invasive aliens and biodiversity in the Mediterranean Sea. Mar Pollut Bull 55: 314–322. 17222869 10.1016/j.marpolbul.2006.11.008

[16] Edelist D, Rilov G, Carlton JT, Spanier E (2013) Restructuring the Sea: profound shifts in the world's most invaded marine ecosystem. Diversity Distrib 19: 69–77.

[17] Seebens H, Gastner MT, Blasius B (2013) The risk of marine bioinvasion caused by global shipping. Ecol Lett 16: 782–790. 23611311 10.1111/ele.12111

[18] Galil BS (2012) Truth and consequences: the bioinvasion of the Mediterranean Sea. Integr Zool 7: 299–311. 22938526 10.1111/j.1749-4877.2012.00307.x

[19] Zenetos Α, Gofas S, Morri C, Rosso A, Violanti D, et al (2012) Alien species in the Mediterranean Sea by 2012. A contribution to the application of European Union's Marine Strategy Framework Directive (MSFD). Part 2. Introduction trends and pathways. Medit Mar Sci 13: 328–352.

[20] Hulme PE, Bacher S, Kenis M, Klotz S, Kühn I, et al (2008) Grasping at the routes of biological invasions: a framework for integrating pathways into policy. J Appl Ecol 45: 403–414.

[21] Drake JM, Lodge DM (2004) Global hot spots of biological invasions: evaluatingoptions for ballast-water management. Proc R Soc Lond B Biol Sci 271: 575–580. 10.1098/rspb.2003.2629 PMC1691629 15156914

[22] Thomas VG, Vasarhelyi C, Niimi AJ (2009) Legislation and capacity for rapid-response management of nonindigenous species of fish in contiguous waters of Canada and the USA. Aquatic Conserv: Mar Freshw Ecosyst 19: 354–364.

[23] Pang K, Martindale MQ (2008) *Mnemiopsis leidyi* spawning and embryo collection. CSH Protoc 2008: pdb. Prot 5085. 10.1101/pdb.prot5085 21356725

[24] Vinogradov ME, Shushkina EA, Musaeva EI, Sorokin PY (1989) Ctenophore *Mnemiopsis leidyi* (A. Agassiz) (Ctenophora, lobata): new settlers in the Black Sea. Oceanology 29: 293–299.

[25] Kideys AE, Niermann U (1994) Occurrence of *Mnemiopsis* along the Turkish coasts (from northeastern Mediterranean to Istanbul). ICES J Mar Sci 51: 423–427.

[26] Shiganova TA, Mirzoyan ZA, Studenikina EA, Volovik SP, Siokou-Frangou I, et al (2001) Population development of the invader ctenophore *Mnemiopsis leidyi* in the Black Sea and other seas of the Mediterranean basin. Mar Biol 139: 431–445.

[27] Ivanov VP, Kamakin AM, Ushivtsev VB, Shiganova TA, Zhukova OP, et al (2000) Invasion of the Caspian Sea by the comb jellyfish *Mnemiopsis leidyi* (Ctenophora). Biol Invas 2: 255–258.

[28] Boero F, Putti M, Trainito E, Prontera E, Piraino S, et al (2009) First records of *Mnemiopsis leidyi* (Ctenophora) from the Ligurian, Thyrrhenian and Ionian Seas (Western Mediterranean) and first record of *Phyllorhiza punctata* (Cnidaria) from the Western Mediterranean. Aquat Invas 4: 675–680.

[29] Fuentes VL, Atienza D, Gili JM, Purcell JE (2009) First record of *Mnemiopsis leidyi* A. Agassiz 1865 off the NW Mediterranean coast of Spain. Aquat Invas 4: 671–674.

[30] Galil B, Kress N, Shiganova TA (2009) First record of *Mnemiopsis leidyi* A. Agassiz, 1865 (Ctenophora; Lobata; Mnemiidae) off the Mediterranean coast of Israel. Aquat Invas 4: 356–362.

[31] Shiganova TA, Malej A (2009) Native and non-native ctenophores in the Gulf of Trieste, Northern Adriatic Sea. J Plank Res 31: 61–71.

[32] Reusch TBH, Bolte S, Sparwel M, Moss AG, Javidpour J (2010) Microsatellites reveal origin and genetic diversity of Eurasian invasions by one of the world's most notorious marine invader, *Mnemiopsis leidyi* (Ctenophora). Mol Ecol 19: 2690–2699. 20561193 10.1111/j.1365-294X.2010.04701.x

[33] Ghabooli S, Shiganova TA, Zhan AB, Cristescu ME, Eghtesadi-Araghi P, et al (2011) Multiple introductions and invasion pathways for the invasive ctenophore *Mnemiopsis leidyi* in Eurasia. Biol Invasions 13: 679–690.

[34] Bolte S, Fuentes V, Haslob H, Huwer B, Thibault-Botha D, et al. (2013) Population genetics of the invasive ctenophore *Mnemiopsis leidyi* in Europe reveal source-sink dynamics and secondary dispersal to the Mediterranean Sea. Mar Ecol Prog Ser doi: 10.3354/meps10321.

[35] Elphinstone MS, Hinten GN, Anderson MJ, Nock CJ (2003) An inexpensive and high-throughput procedure to extract and purify total genomic DNA for population studies. Mol Ecol Notes 3: 317–320.

[36] White TJ, Burns T, Lee S, Taylor J (1990) Amplification and direct sequencing of fungal ribosomal RNA genes for phylogenetics. In: Innis MA, Gelfand DH, Shinsky JJ, White TJ, editors. PCR Protocols: A Guide to Methods and Applications. Academic Press, San Diego, pp. 315–322.

[37] Nei M (1987) Molecular evolutionary genetics. Columbia University Press, New York

[38] Librado P, Rozas J (2009) DnaSP v5: A software for comprehensive analysis of DNA polymorphism data. Bioinformatics 25: 1451–14. 19346325 10.1093/bioinformatics/btp187

[39] Tamura K, Dudley J, Nei M, Kumar S (2007) MEGA4: Molecular Evolutionary Genetics Analysis (MEGA) Software Version 4.0. Mol Biol Evol 24: 1596–1599. 17488738 10.1093/molbev/msm092

[40] Clement M, Posada D, Crandall KA (2000) TCS: a computer program to estimate gene genealogies. Mol Ecol 9: 1657–1659. 11050560 10.1046/j.1365-294x.2000.01020.x

[41] Excoffier L, Guillaume L, Schneider S (2005) Arlequin (version 3.0): An integrated software package for population genetics data analysis. Evol Bioinform 1: 47–50. PMC2658868 19325852

[42] Gotelli NJ, Entsminger GL (2006) Ecosim: Null Models Software for Ecology, Version 7. Acquired Intelligence Inc. and Kesey-Bear, Jericho, VT 05465. Available at: http://garyentsminger.com/ecosim.htm.

[43] Chao A, Shen TJ (2003) User's Guide for Program SPADE (Species Prediction And Diversity Estimation) Updated August 2008. Available at: http://chao.stat.nthu.edu.tw/.

[44] Chao A, Shen TJ (2006) SPADE Version 3.1. Available at: http://chao.stat.nthu.edu.tw/.

[45] Roman J, Darling JA (2007) Paradox lost: genetic diversity and the success of aquatic invasions. Trends Ecol Evol 22: 454–464. 17673331 10.1016/j.tree.2007.07.002

[46] Harbison GR, Miller RL (1986) Not all ctenophores are hermaphrodites. Studies on the systematics, distribution, sexuality and development of the two species of *Ocyrosis* . Mar Biol 90: 413–424.

[47] Facon B, Genton BJ, Shykoff J, Jarne P, Estoup A, et al (2006) A general eco-evolutionary framework for understanding bioinvasions. Trends Ecol Evol 21: 130–135. 16701488 10.1016/j.tree.2005.10.012

[48] Woods EC, Hastings AP, Turley NE, Heard SB, Agrawal AA (2012) Adaptive geographical clines in the growth and defense of native plant. Ecol Monogr 82: 149–168.

[49] Goldstien SJ, Inglis GJ, Schiel DR, Gemmell NJ (2013) Using temporal sampling to improve attribution of source populations for invasive species. PloS One 8: doi: 10.1371/journal.pone.0065656. 10.1371/journal.pone.0065656 PMC3670837 23755264

[50] Zamud KR, Savage WK (2003) Historical isolation, range expansion and secondary contact of two highly divergent mitochondrial lineages in spotted salamanders (*Ambystoma maculatum*). Evolution 57: 1631–1652. 12940367 10.1554/02-342

[51] Shiganova TA, Dumont HJ D, Sokolsky AF, Kamakin AM, Tinentova D, et al (2004) Population dynamics of *Mnemiopsis leidyi* in the Caspian Sea, and effects on the Caspian ecosystem. In: Dumont H, Shiganova TA, Niermann U, editors. The Ctenophore *Mnemiopsis leidyi* in the Black, Caspian and Mediterranean Seas and other aquatic invasions. Kluwer Academic Publication, Dordrecht. pp. 71–111.

